# Stress-Inducible Transcription Factor NUPR1 Is Involved in the Inhibitory Effects Exerted by Statins on Insulin Action in ER-Positive Breast Cancer Cells

**DOI:** 10.3390/cells15030284

**Published:** 2026-02-02

**Authors:** Domenica Scordamaglia, Azzurra Zicarelli, Francesca Cirillo, Marianna Talia, Ernestina Marianna De Francesco, Roberta Malaguarnera, Marcello Maggiolini, Rosamaria Lappano

**Affiliations:** 1 Department of Medicine and Surgery, “Kore” University of Enna, 94100 Enna, Italy; domenica.scordamaglia@unikore.it (D.S.); azzurra.zicarelli@unikore.it (A.Z.); francesca.cirillo@unikore.it (F.C.); ernastinamarianna.defrancesco@unikore.it (E.M.D.F.); roberta.malaguarnera@unikore.it (R.M.); 2 Department of Pharmacy, Health and Nutritional Sciences, University of Calabria, 87036 Rende, Italy; marianna.talia@unical.it; 3 Department of Experimental and Clinical Medicine, University “Magna Græcia” of Catanzaro, 88100 Catanzaro, Italy

**Keywords:** breast cancer, statins, BCAHC-1 cells, insulin, insulin receptor

## Abstract

Obesity is frequently associated with metabolic alterations like hypercholesterolemia and hyperinsulinemia and represents a major risk factor for several diseases, including breast cancer (BC). Insulin signaling, as well as the frequent overexpression of the insulin receptor (IR), play a key role in BC progression. Emerging evidence suggests that the widely prescribed lipid-lowering drugs, named statins, may reduce the risk of recurrence and blunt BC cell proliferation, mainly inhibiting the HMGCR-dependent activation of the mevalonate pathway. In this study, we investigated the effects of simvastatin, atorvastatin and rosuvastatin in BC cells stimulated by insulin. To this end, we used as a BC model system MCF7 cells and naturally immortalized BCAHC-1 cells, which are characterized by high IR-expression levels. Our investigation demonstrates that statins reduce the proliferation and clonogenic capacity of BC cells prompted by insulin treatment. Mechanistically, statins impair the IR-mediated signaling and downregulate the stress-inducible transcription factor NUPR1, a known regulator of cancer progression. Importantly, NUPR1 inhibition blunted the stimulatory action of insulin on BC cells. Consistent with these findings, survival analyses of large cohorts of patients revealed that high levels of NUPR1 are associated with poor BC prognosis. Overall, our results provide novel mechanistic evidence supporting the repositioning of statins in BC, particularly in tumors characterized by elevated IR expression and activity.

## 1. Introduction

Breast cancer (BC) remains the most frequently diagnosed malignancy among women worldwide and constitutes a leading cause of cancer-related mortality [1]. Although advancements in early detection and the development of targeted therapies have significantly improved the outcomes of patients, several metabolic and systemic conditions, including obesity, hyperinsulinemia, and hypercholesterolemia, increase BC risk, aggressive features and resistance to therapeutics [2,3]. Recent evidence indicates that obesity affects approximately 20–40% of all BC cases, particularly in postmenopausal women, through the increase in hormones and growth factors levels, together with the establishment of a chronic, low-grade inflammatory state [4,5,6,7]. In this regard, it should be noted that elevated insulin levels have been correlated with BC incidence, recurrence, tumor growth and survival [8,9,10,11]. Insulin acts as a potent mitogenic hormone by binding to the insulin receptor (IR), thereby promoting cell cycle progression, inhibiting apoptosis and cooperating with estrogen signaling pathways toward cell proliferation [12,13,14,15]. Moreover, IR is frequently overexpressed in BC cells and associated with enhanced tumor growth and metastasis [16,17]. Next, the activation of IR by insulin triggers the recruitment and phosphorylation of insulin receptor (IR) substrates, thus initiating downstream signaling cascades such as the PI3K/AKT/mTOR and RAS/MAPK pathways. These signaling events orchestrate extensive transcriptional reprogramming that in turn promotes cell growth, survival, metabolic adaptation, and angiogenesis [12,13,14,18,19]. Notably, alterations of these signaling cascades in BC are frequently associated with enhanced metastatic potential and the development of resistance to anticancer therapies [20,21,22,23].

These signaling networks orchestrate extensive transcriptional reprogramming that promotes cell growth, survival, metabolic adaptation, and angiogenesis [12,13,14,18,19]. Notably, dysregulation of these pathways in breast cancer is frequently associated with enhanced metastatic potential and the development of resistance to anticancer therapies, underscoring the critical role of aberrant insulin signaling in tumor progression and treatment failure.

Hypercholesterolemia is frequently observed in individuals with obesity and insulin resistance and is associated with increased tumor cell proliferation, enhanced membrane fluidity, elevated steroid hormone synthesis and tumor invasiveness [24,25,26]. In addition, cholesterol-derived metabolites, particularly oxysterols, have been shown to function as selective estrogen receptor modulators (SERMs) [27,28,29]. These molecules can bind to and activate the estrogen receptor (ER)α, thereby mimicking estrogenic signaling and promoting BC progression [30,31,32,33]. Accordingly, pharmacological agents that regulate cholesterol homeostasis have been demonstrated to exert anti-tumor effects by disrupting key metabolic and signaling pathways involved in BC development. Statins, originally developed and currently employed as lipid-lowering drugs, have emerged as potential protective agents in metabolically dysregulated BC patients. Statins act as competitive inhibitors of 3-hydroxy-3-methylglutaryl-coenzyme A reductase (HMGCR), the rate-limiting enzyme in the mevalonate pathway, which is essential for endogenous cholesterol biosynthesis [34]. Beyond their lipid-lowering properties, statins exhibit anti-inflammatory, antioxidant, and immunomodulatory effects, which may contribute to their anticancer potential, including impaired cancer cell viability and reduced tumor progression [35,36,37,38]. Preclinical studies have also shown that statins can suppress BC growth, recurrence and metastasis, particularly in ER-positive BC patients with elevated cholesterol levels [39,40,41]. The beneficial effects of statins appear to be mediated not only by modulation of cholesterol metabolism but also by their capability to counteract cancer-promoting inflammatory responses and to inhibit the prenylation of oncogenic proteins such as RAS and Rho, which are key regulators of cell proliferation and survival [42,43,44,45,46]. Despite variability in clinical outcomes across studies, statins represent a promising class of repurposed agents for BC treatment, owing to their favorable safety profile and widespread clinical use [37]. Moreover, combination strategies including statins and standard therapies are currently under investigation for the improvement of therapeutic responses. In this regard, ongoing clinical trials are evaluating combination therapies, although further studies are needed to identify optimal treatment strategies for BC patients with coexisting metabolic disorders NCT03324425, NCT05464810 [47].

Here, we provide evidence regarding novel mechanisms by which statins, such as simvastatin, atorvastatin and rosuvastatin, inhibit the proliferative effects induced by insulin on either the naturally immortalized BC cell line BCAHC-1, which is characterized by high IR levels, or the well-known IR-positive MCF7 BC cells [48,49]. Of note, we show for the first time that the stress-responsive transcription factor nuclear protein 1 (NUPR1) is involved in the anticancer action of statins, therefore providing novel mechanistic insights regarding the inhibitory effects of statins in BC.

## 2. Materials and Methods

### 2.1. Cell Cultures

BCAHC-1 cells were isolated and characterized as previously described [12], deposited at the Leibniz Institute DSMZ–German Collection of Microorganisms and Cell Cultures (DSMZ) and protected by patent (n. 102019000022167). MCF7 cells were provided by ATCC (Manassas, VA, USA), used within six months after resuscitation, and routinely tested and authenticated according to ATCC guidelines. Both cell lines were cultured in DMEM/F-12 medium containing phenol red and supplemented with 5% fetal bovine serum (FBS) and 100 μg/mL penicillin/streptomycin. Cells were regularly passaged and expanded under standard conditions at 37 °C in a humidified atmosphere 5% CO_2_.

### 2.2. Reagents

Insulin was solubilized in water and used at a final concentration of 10 nM, in accordance with previous studies [12,13,14,50,51,52]. The NUPR1 inhibitor zzw-115, simvastatin, atorvastatin, and rosuvastatin were solubilized in dimethyl sulfoxide (DMSO, 0.1%), which served as the control vehicle for all the experiments. According to previous investigations [53,54,55], statins were used at a final concentration of 1 μM, and zzw-115 was used at 10 nM. All reagents were purchased from Merck Life Science (Milan, Italy) except for zzw-115, which was purchased from Selleck Chemicals (Houston, TX, USA).

### 2.3. Gene Expression Studies

Total RNA was isolated and reverse-transcribed into cDNA as previously described [56]. The expression levels of selected genes were determined by real-time PCR using the QuantStudio^TM^ 7 Flex Real-Time PCR System (Thermo Fisher Scientific, Milan, Italy). Gene-specific primers were designed using Primer Express software version 3.0 (Applied Biosystems): 5′-TGACCTCTATAGCCTGGCCC-3′ (*NUPR1* forward) and 5′-CACCTCCTGTAACCAAGGCA-3′ (*NUPR1* reverse); 5′-AAGCCACCCCACTTCTCTCTAA-3′ (*ACTB* forward) and 5′-CACCTCCCCTGTGTGGACTT-3′ (*ACTB* reverse). Assays were performed in triplicate, and results were normalized to actin beta (*ACTB*) expression. Relative RNA expression levels were calculated as fold changes using the comparative Ct (ΔΔCt) method. Briefly, ΔCt values were obtained by subtracting the Ct of ACTB from that of the target gene, and ΔΔCt values were calculated relative to vehicle-treated samples. Values obtained from vehicle-treated cells were set to one-fold induction, and treatment-induced activity was calculated accordingly. Fold changes in gene expression were determined using the formula 2^−ΔΔCt^ and are presented relative to control conditions.

### 2.4. Western Blot Analysis

Cells were cultured in 10 cm dishes, subjected to the indicated treatments, and subsequently lysed in 500 μL of RIPA buffer supplemented with protease and phosphatase inhibitors (1.7 mg/mL aprotinin, 1 mg/mL leupeptin, 200 mmol/L phenylmethylsulfonyl fluoride, 200 mmol/L sodium orthovanadate, and 100 mmol/L sodium fluoride). Cells were treated for 18 h with statins, and then exposed to insulin for 30 min or 6 h, according to previous investigations [11,14,52,53,57,58]. Samples were then centrifuged at 13,000 rpm for 10 min and protein concentrations were determined using the BCA protein assay according to the manufacturer’s instructions (Thermo Fisher Scientific, Milan, Italy). Equal amounts of whole-protein extract were resolved on an 8% or 10% SDS-polyacrylamide gel and transferred to nitrocellulose membranes (Merck, Milan, Italy), which were probed with primary antibodies against IR (N-20) (sc-710, 1:1000) and IGF1R (7G11) (sc-81464, 1:1000) (Santa Cruz Biotechnology, DBA, Milan, Italy), IRS1 (A12569, 1:1000) (Antibodies, Sial, Rome, Italy), AKT (H-136, sc-8312, 1:1000) (Santa Cruz Biotechnology, DBA, Milan, Italy), pIR (Tyr1146) (3021, 1:1000) (Cell Signaling Technology, Euroclone, Milan, Italy), pIRS1 Tyr896 (A93833, 1:1000) (Antibodies, Sial, Rome, Italy), pAKT Ser473 (D9E, 1:1000) (Cell Signaling Technology, Euroclone, Milan, Italy), NUPR1 (NBP1-98280, 1:750) (Novus Biologicals, Bio-Techne SRL, Milan, Italy), and β-actin (AC-15, 1:4000) (Santa Cruz Biotechnology, DBA, Milan, Italy). Proteins were detected by horseradish peroxidase-linked secondary antibodies (1:10,000) (Bio-Rad, Milan, Italy) and then revealed using the chemiluminescent substrate for Western blotting Clarity Western ECL Substrate (Bio-Rad, Milan, Italy). Protein levels were quantified by densitometric analysis using ImageJ software. For each sample, the intensity of the band corresponding to the protein of interest was measured within an identical region of interest (ROI). The background signal was subtracted using an adjacent area lacking a specific signal. The background-corrected signal was then normalized to the corresponding loading control (e.g., total protein or housekeeping protein) detected on the same membrane, to account for potential differences in protein loading and transfer efficiency. Finally, the integrated density was calculated as the ratio between the protein of interest and the corresponding loading control.

### 2.5. Proliferation Assay

Cells (1 × 10^4^) were seeded in 24-well plates in complete growth medium, incubated in medium supplemented with 5% charcoal-stripped FBS, and then treated as indicated, in accordance with previous investigations [12,13,14,52]. Treatments were refreshed daily, while the culture medium was replaced every 2 days. Cell proliferation was assessed on day 5 using the trypan blue exclusion method prior to direct cell counting using the Countess Automated Cell Counter, according to the manufacturer’s instructions (Thermo Fisher Scientific, Milan, Italy).

### 2.6. Colony Formation Assay

Cells were grown in standard culture conditions until approximately 90% confluence, then trypsinized, counted and seeded at a density of 1 × 10^3^ per well in six-well plates containing medium supplemented with 5% charcoal-stripped FBS. Cells were exposed to statins or zzw-115 1 h before insulin exposure. Treatments were renewed together with the medium every 2 days. According to previous investigations [12,13,14], after 10 days, colonies were washed with PBS, fixed with acetone:methanol (1:1) for 3 min at room temperature and stained with 0.1% Crystal Violet (Merck, Milan, Italy) for 20 min. For each condition, 10 images were acquired using a digital camera, and colony numbers were quantified using the ImageJ software.

### 2.7. Spheroid Formation Assay

Three-dimensional spheroids were generated by seeding 100 μL of a single-cell suspension (1 × 10^4^ cells/well) into 96-well plates pre-coated with 2% agarose to prevent cell adhesion and promote spheroid formation. Three days after seeding, 50% of the medium was replaced every 2 days with fresh medium containing the appropriate treatments. When applicable, transfections were also renewed at the same intervals. On day 10, spheroids were imaged using the Cytation 3 Cell Imaging Multimode Reader, as recommended by the manufacturer’s protocol (BioTek, AHSI, Milan, Italy). Spheroid area was quantified using Gen5 software (BioTek, AHSI, Milan, Italy), which allows for automated image acquisition and analysis.

### 2.8. Data Sources

Gene expression and clinical data were obtained from The Cancer Genome Atlas (TCGA) and the Molecular Taxonomy of Breast Cancer International Consortium (METABRIC) [59,60]. TCGA RNA-Seq V2 RSEM data from the Invasive Breast Cancer Cohort (n = 1247) were retrieved from the UCSC Xena platform (https://xenabrowser.net/). Data were accessed on 5 April 2025. Only primary tumor samples were retained (n = 1101), based on the “sample type” annotation. METABRIC gene expression data (log_2_-transformed microarray intensities) and clinical information (n = 2509) were retrieved from the cBioPortal for Cancer Genomics (http://www.cbioportal.org/) on 15 July 2025. In both datasets, samples were stratified by ER status determined by immunohistochemistry. Gene expression and clinical data were filtered to exclude samples with missing values.

### 2.9. Survival Analysis

Survival analyses were performed using NUPR1 expression data and clinical outcomes from ER-positive BC patients of the METABRIC and TCGA cohorts. Overall survival data were used for METABRIC, while disease-free interval data were used for TCGA. METABRIC samples were filtered based on vital status, excluding the patients classified as “died of other causes”. The survivALL package was employed to examine Cox proportional hazards for all possible points of separation (low-high cut-points), selecting the cut-point with the lowest *p*-value [61]. Therefore, patients were stratified into high and low NUPR1 expression groups. Kaplan–Meier survival curves were generated using the survival (version 3.8.3) and the survminer (version 0.5.0) R packages.

### 2.10. Correlation and Pathway Analysis

Pearson correlation coefficients (*r* values) between NUPR1 expression levels and those of all the other genes were computed in the TCGA cohort of ER-positive BC patients using the cor.test() function in R Studio (version 2024.04.2+764). The *t*-test was employed to evaluate the statistical relevance of the measurements and a *p* value < 0.05 was considered as a significant threshold. Subsequently, the 500 genes exhibiting the highest positive correlation coefficients were subjected to Kyoto Encyclopedia of Genes and Genomes (KEGG) pathway enrichment analysis using the Database for Annotation, Visualization and Integrated Discovery (DAVID) functional annotation tool (https://davidbioinformatics.nih.gov/) on 30 July 2025, with Homo sapiens selected as the background species.

### 2.11. Statistical Analysis

Statistical analyses were performed using ANOVA followed by Newman–Keuls’ test to determine differences in means. (*) indicates *p* < 0.05, (**) indicates *p* < 0.005, (***) indicates *p* < 0.0005, (****) indicates *p* < 0.0001.

## 3. Results

### 3.1. Statins Inhibit the Insulin-Stimulated Proliferation of BC Cells and the Activation of Insulin/IR Transduction Signaling

**Figure 1:**
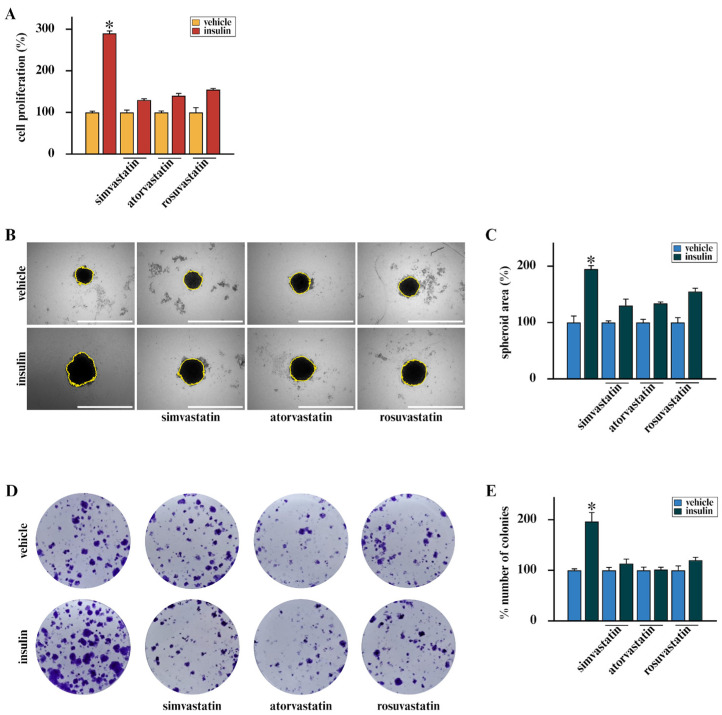
Statins inhibit the mitogenic action of insulin in BCAHC-1 cells. (**A**) Proliferation of BCAHC-1 cells was evaluated after 5 days of treatment with vehicle or 10 nM insulin, administered alone or in combination with 1 μM simvastatin, atorvastatin or rosuvastatin. Values of cells treated with vehicle were set as 100%, upon which proliferation induced by treatments was determined. (**B**) Representative images of spheroids (a single spheroid/well) from BCAHC-1 spheroid cultures grown on agar-coated plates and exposed for 10 days to vehicle or 10 nM insulin alone or in combination with 1 μM simvastatin, atorvastatin or rosuvastatin. Scale bar: 1000 μm. (**C**) Quantification of spheroid growth. Values of vehicle-treated BCAHC-1 cells were set as 100%, upon which spheroid growth was determined. (**D**) Colony formation assay in BCAHC-1 cells exposed to vehicle or 10 nM insulin alone or in combination with 1 μM simvastatin, atorvastatin or rosuvastatin. After 10 days of incubation, cell colonies were stained and pictures were captured by a digital camera. (**E**) Colonies were counted using the ImageJ program (version 1.54p). Values represent the mean ± SD of three independent experiments performed in triplicate. (*) indicates *p* < 0.05.

On the basis of the stimulatory action of insulin in BC [11,13,14,46] and considering that approximately 80% of BCs display high IR expression [13,47,48], we aimed at exploring novel strategies to counteract the mitogenic action of insulin signaling. In this vein, we employed BCAHC-1 cells, a peculiar BC cellular model characterized by high expression of IR and the absence of the insulin-like growth factor 1 receptor (IGF1R), as we have previously demonstrated [12] and as shown in Supplementary Figure S1. In addition, we used the well-known IR-positive MCF7 BC cells [49,50]. Considering that several clinical studies have indicated the ability of statins to reduce the recurrence of BC [62,63,64], we aimed to provide novel evidence regarding their mechanisms of action in BC cells. Remarkably, we ascertained that simvastatin, atorvastatin, and rosuvastatin inhibit the growth (Figure 1A) and the colony-forming ability of BCAHC-1 cells (Figure 1D,E), as well as the spheroid expansion of both BCAHC-1 (Figure 1B,C) and MCF7 (Supplementary Figure S2A,B) BC cells triggered by insulin stimulation.

**Figure 2:**
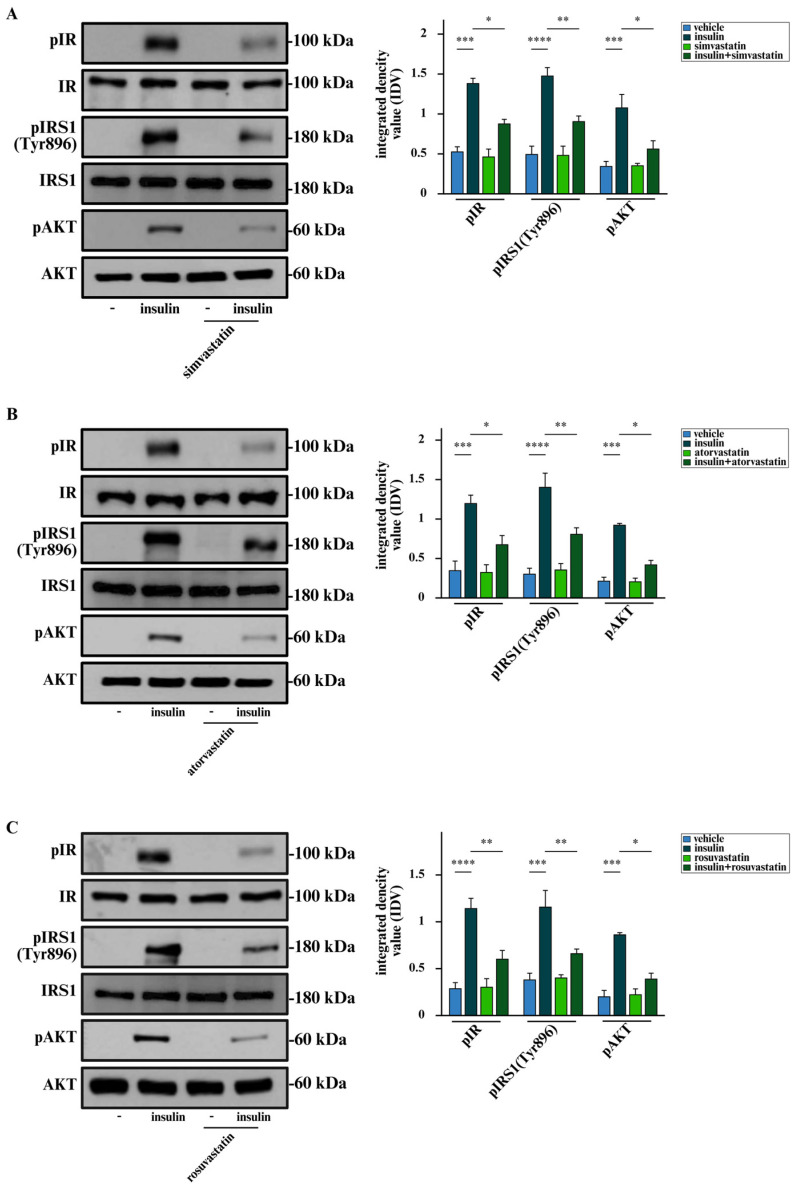
Statins interfere with the activation of IR signaling triggered by insulin in BCAHC-1 cells. Protein levels of phosphorylated IR (pIR), IRS1 Tyr896 (pIRS1 Tyr896) and AKT (pAKT) in BCAHC-1 cells exposed for 30 min to vehicle or 10 nM insulin in the presence or absence of 1 µM simvastatin (**A**), atorvastatin (**B**) or rosuvastatin (**C**), as indicated. Side panels show integrated density values (IDVs) calculated as the ratio of the protein of interest to IR, IRS1, and AKT, which served as loading controls, as indicated. Values represent the mean ± SD of three independent experiments performed in triplicate. (*) indicates *p* < 0.05, (**) indicates *p* < 0.005, (***) indicates *p* < 0.0005, (****) indicates *p* < 0.0001.

In order to characterize the molecular mechanisms by which statins prevent the stimulatory effects elicited by insulin, we evaluated their potential to interfere with the IR transduction pathway. In this regard, we found that simvastatin, atorvastatin and rosuvastatin decrease the insulin-induced phosphorylation of IR, without affecting its expression levels (Figure 2A–C). Additionally, statins prevented the insulin-stimulated activation of two downstream effectors of IR, such as IRS1 and AKT (Figure 2A–C).

### 3.2. Statins Prevent the Insulin-Induced Increase in NUPR1 in BC Cells

**Figure 3:**
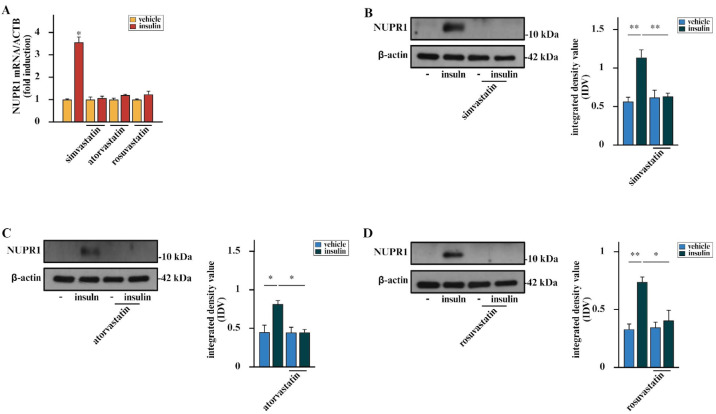
Statins inhibit the insulin-induced up-regulation of NUPR1 in BCAHC-1 cells. (**A**) mRNA levels of NUPR1 evaluated by real-time PCR in BCAHC-1 cells exposed for 6 h to vehicle or 10 nM insulin alone or in combination with 1 μM simvastatin, atorvastatin or rosuvastatin, which were added to the culture medium 18 h before vehicle or insulin exposure. Values are normalized to the actin beta (ACTB) expression and presented as fold changes in mRNA expression upon treatments compared to vehicle. Immunoblot of NUPR1 from BCAHC-1 cells treated for 6 h with vehicle or 10 nM insulin alone or in combination with 1 μM simvastatin (**B**), atorvastatin (**C**) or rosuvastatin (**D**) that were added to the culture medium 18 h before the treatment with vehicle or insulin. Side panels show integrated density value (IDV) calculated by the ratio between NUPR1 and β-actin, which served as the loading control. Values represent the mean ± SD of three independent experiments performed in triplicate. (*) indicates *p* < 0.05, (**) indicates *p* < 0.005.

Our previous study showed that in BCAHC-1 cells, insulin increases the expression of various genes involved in BC cell proliferation, including the transcriptional regulator NUPR1 [12]. Also referred to as p8 or Com-1, NUPR1 contributes to the regulation of DNA repair and chromatin remodeling, endoplasmic reticulum stress responses, oxidative stress activity, cell cycle machinery, autophagy, apoptosis and ferroptosis arrest [65,66,67,68]. Recent investigations have also indicated that ER-positive BC cells, including those resistant to endocrine therapy (ET), exhibit high levels of NUPR1 that may contribute to the stimulation of BC cell growth [58,59]. On these bases and considering that a negative correlation between statins and the proliferative rate of BC cells has been previously reported [60], we aimed to determine whether statins could regulate the expression of NUPR1 in BCAHC-1 and MCF7 BC cells. Remarkably, we assessed that the up-regulation of NUPR1 by insulin is inhibited in the presence of simvastatin, atorvastatin, and rosuvastatin at both mRNA (Figure 3A, Supplementary Figure S2C) and protein levels (Figure 3B–D, Supplementary Figure S2D–F) in both BC cell types.

### 3.3. NUPR1 Expression Correlates with Poor Prognosis in BC Patients and Its Inhibition Prevents the Growth of BC Cells Prompted by Insulin

**Figure 4:**
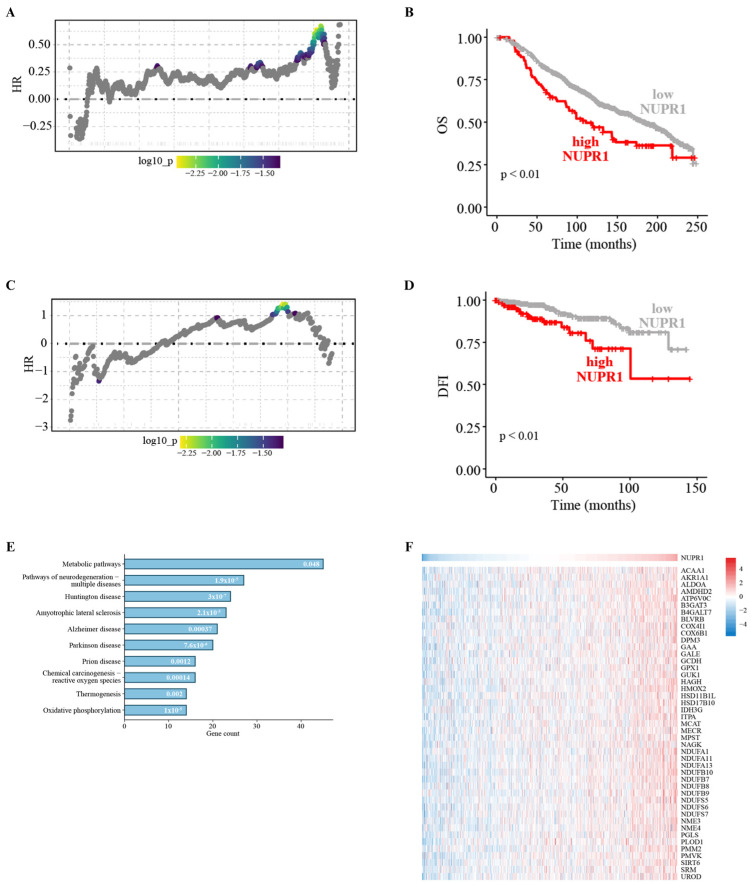
NUPR1 is correlated with poor outcomes in ER-positive BC patients. The plotALL function was used to calculate hazard ratios (HR, y-axis) across all possible cut-points of NUPR1 expression in breast cancer (BC) patients from the METABRIC (**A**) and TCGA (**C**) datasets. Patients are ranked along the x-axis by increasing NUPR1 expression. The color bar gradient stands for the range of the most significant points of separation of the population (low–high significance = blue–yellow gradient) based on NUPR1 expression and survival rates of each patient. Kaplan–Meier curves showing the correlation between NUPR1 expression and overall survival (OS) in the METABRIC cohort (**B**) and disease-free interval (DFI) in the TCGA cohort (**D**) of ER-positive BC patients. (**E**) Bar plot displaying the top ten most significant enriched NUPR1-associated pathways identified by KEGG pathway analysis. The number of genes included in each pathway is displayed along the x-axis, while the different KEGG terms are shown along the y-axis. *p*-values are annotated within each bar. *p* < 0.05 was set as a significant threshold. (**F**) Heatmap showing the expression of genes belonging to the KEGG term “metabolic pathways” and correlated to NUPR1 gene levels in ER-positive BC patients from the TCGA dataset. Samples were ordered from left to right by increasing NUPR1 expression; red indicates high expression and blue indicates low expression.

**Figure 5:**
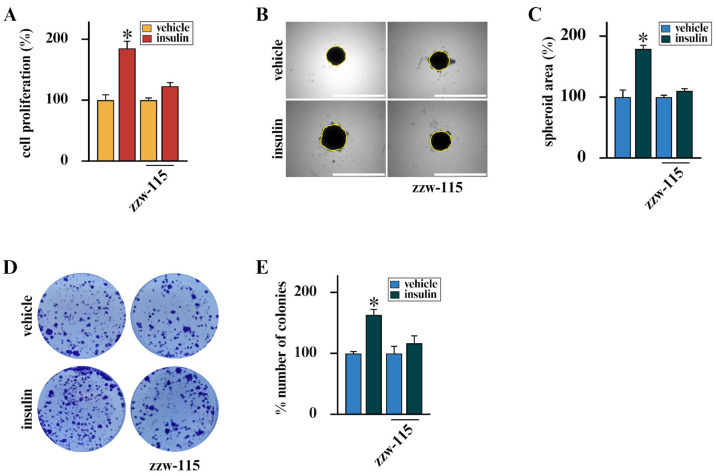
The NUPR1 inhibitor zzw-115 interferes with the proliferative activity in BCAHC-1 cells. (**A**) Proliferation of BCAHC-1 cells after 5 days treatment with vehicle or 10 nM insulin alone or in combination with 10 nM zzw-115. (**B**) Representative pictures of spheroids (a single spheroid/well) from the BCAHC-1 spheroid cultures grown on agar-coated plates and exposed for 10 days to vehicle or 10 nM insulin alone or in combination with 10 nM zzw-115. Scale bar: 1000 μm. (**C**) Quantification of spheroid growth. Values of vehicle-treated BCAHC-1 cells were set as 100%, upon which spheroid growth was determined. (**D**) Colony formation assay in BCAHC-1 cells exposed to vehicle or 10 nM insulin alone or in combination with 10 nM zzw-115. After 10 days of incubation, cell colonies were stained and pictures were captured by a digital camera. Colonies were counted using the program WCIF ImageJ for Windows. (**E**) Values represent the mean ± SD of three independent experiments performed in triplicate. (*) indicates *p* < 0.05.

**Figure 6:**
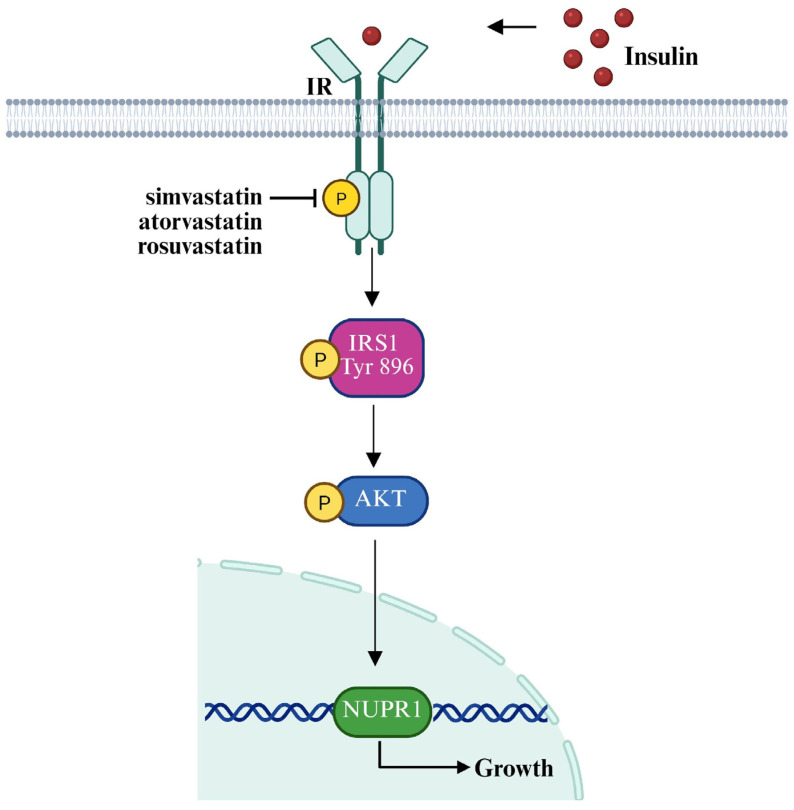
Cartoon depicting the statin-mediated inhibition of insulin action in BC cells. Created with BioRender.com.

In order to explore the clinical significance of NUPR1 expression in BC, we performed bioinformatics analyses on cohorts of ER-positive BC patients, taking into account that BCAHC-1 cells express the ERα 46 kDa isoform [12,13] and MCF7 cells express the full-length 66kDa ERα [61]. Interestingly, survival analyses indicated that NUPR1 expression correlates with poor outcomes in ER-positive BC patients of the METABRIC and the TCGA datasets (Figure 4A–D). To gain mechanistic insights into this association, we interrogated the TCGA cohort of ER-positive BC patients, ranking the first 500 genes positively correlated with NUPR1 in accordance with their Pearson correlation coefficient. Hence, to elucidate the biological processes associated with NUPR1 co-expression, KEGG pathway enrichment analysis was conducted using DAVID. The top 500 genes most strongly and positively correlated with NUPR1 were found to be enriched in several pathways, as schematically illustrated in Figure 4 (panel E). Among these, the term “metabolic pathways” included the largest number of genes, whose expression patterns are shown in the heatmap of Figure 4 (panel E). In accordance with the recognized role of NUPR1 as a regulator of metabolic programs that fuel biomass production and energy supply [65,66,69], we hypothesized that its activity could be crucial for sustaining BC cell growth. Consistent with this hypothesis, we found that the insulin-induced proliferative effects observed in BCAHC-1 and MCF7 cells occur in a NUPR1-dependent manner. In particular, we observed that the selective inhibitor of the nuclear translocation of the transcription factor, namely zzw-115, effectively abolishes the proliferative responses prompted by insulin in both BC cell types (Figure 5A–E, Supplementary Figure S2G,H). These findings support the involvement of the IR/NUPR1 signaling axis in mediating the growth action of insulin in IR-positive BCAHC-1 and MCF7 cells. Taken together, our data offer valuable insights into the promising role of statins within combination therapies designed to counteract the challenges posed by the proliferative effects mediated by the insulin/IR axis in BC cells (Figure 6).

## 4. Discussion

Elevated circulating levels of hormones commonly associated with obesity, type 2 diabetes and metabolic syndrome, particularly insulin, have been related to increased incidence of several types of cancer and higher related mortality [2,70,71,72]. Given the global rise in metabolic diseases like obesity and diabetes, as well as the clinical evidence demonstrating that hyperinsulinemia may contribute to BC therapeutic resistance [73], it remains crucial to elucidate the mechanisms by which insulin triggers cancer progression.

The insulin signaling cascade is initiated by the binding of insulin and insulin-like growth factors (IGFs) to two structurally related tyrosine kinase receptors (IR-A and IR-B). Upon activation, these receptors engage a set of shared intracellular signaling components, such as IRS and PI3K/AKT, among others, leading to relevant downstream effects [74,75]. The involvement of the mitogenic IR-A isoform has been documented in various human malignancies, including BC, where IR-A is frequently overexpressed [48,76,77].

Drug repurposing has emerged as a strategic approach in oncology. The identification of new anticancer therapeutic applications for already available and clinically approved non-oncologic drugs may offer significant advantages over traditional drug discovery, in terms of shorter and cheaper development timelines and a faster transition to clinical use [78,79]. In this regard, statins that are widely used as cholesterol-lowering drugs have attracted increasing attention for their potential anti-cancer properties [80]. In recent years, epidemiological and clinical studies have indicated that statins are associated with a reduced risk of BC recurrence and associated mortality [41,81,82]. With regard to the molecular mechanisms potentially involved in the anti-tumor effects of statins, it has been shown that statins inhibit HMGCR, a key enzyme involved in the regulation of the mevalonate pathway, which is frequently dysregulated in cancer [44,83]. Specifically, the inhibition of HMGCR decreases the mevalonate pathway flux by depleting mevalonate-derived intermediates, especially isoprenoids, which are crucial for the prenylation and functional activation of oncogenic small GTP-binding proteins like RAS and RHO [55,84]. Moreover, statin-mediated blockade of the mevalonate pathway has been demonstrated to induce cell cycle arrest and apoptosis, supporting a direct cytotoxic activity associated with the inhibition of HMGCR [53,85]. In addition, the inhibitory effects of statins on the mevalonate pathway lead to an impairment of the YAP and TAZ transduction system, which plays a central role in cancer cell proliferation and survival [86,87].

In accordance with these findings and additional evidence indicating anti-proliferative, pro-apoptotic, and anti-invasive effects of statins in women with high-grade and invasive BCs as well as in diverse BC models [88,89,90], a correlation between the use of statins and reduced BC recurrence and mortality has been reported [91,92,93]. In line with these observations, our study demonstrates that simvastatin, atorvastatin and rosuvastatin effectively counteract the proliferative effects of insulin in BCAHC-1 and MCF7 BC cells, which were used as model systems. Notably, BCAHC-1 cells deriving from a patient with invasive ductal carcinoma [12] exhibit a predominant expression of IR-A with respect to IR-B and lack IGF1R. As such, BCAHC-1 cells represent a unique, valuable and physiologically relevant model to investigate the contribution of insulin/IR-A signaling to BC development, as well as the potential of statins to modulate this oncogenic pathway. It is worth noting that the anti-proliferative action elicited by statins has been confirmed in IR- and ER-positive MCF7 cells, which represent a widely used human BC cell line derived from a metastatic adenocarcinoma [94,95].

Additionally, we ascertained that simvastatin, atorvastatin and rosuvastatin inhibit the insulin-induced phosphorylation of IR, thereby attenuating downstream signaling events, including the activation of IRS1 and Akt. These data indicate that statins interfere with key nodes of the insulin/IR-A signaling cascade, potentially impairing mitogenic and survival signals mediated by this pathway. Furthermore, we found that insulin stimulation leads to a marked up-regulation of NUPR1, a transcription factor acting in response to multiple internal and external stress signals, including DNA damage, endoplasmic reticulum and oxidative stress, inflammation and metabolic stress [69]. Although the role exerted by NUPR1 in cancer is complex and tissue-dependent, previous evidence has indicated an oncogenic function for this stress-responsive gene [66]. In BC, NUPR1 is frequently up-regulated and activates autophagy to promote tumor invasion and metastasis [68,96,97]. Additionally, NUPR1 has recently been indicated as a transcriptional coregulator involved in the maintenance of tamoxifen resistance by physically interacting with the ER-encoding gene in BC cells [98]. In line with these findings suggesting a role for NUPR1 in promoting aggressive features in BC, we observed that the insulin-induced proliferative advantage in BC cells is prevented using the NUPR1 inhibitor zzw-115. Corroborating these data, our integrated bioinformatics analysis in ER-positive BC datasets revealed that the mRNA levels of NUPR1 are associated with worse survival rates, as well as with the expression of diverse genes implicated in the regulation of cellular metabolism. These findings suggest that insulin not only activates proliferative transduction pathways, but also promotes a NUPR1-dependent transcriptional reprogramming that may facilitate an aggressive BC phenotype, which can be counteracted by statins. Nevertheless, further studies are warranted for a deeper evaluation of the mechanisms underlying the capability of statins to prevent the stimulatory action of insulin/IR-A signaling in BC. In particular, it would be interesting to explore the potential of statins to (i) sequester insulin via the up-regulation of the Insulin-like Growth Factor Binding Protein 7 (IGFBP-7), which acts as an insulin sequestering protein, thereby reducing insulin availability [99]; (ii) down-regulate molecular chaperones involved in IR trafficking to the plasma membrane; (iii) stimulate the activity of a negative regulator of insulin signaling named Protein Tyrosine Phosphatase 1B (PTPN1), which is a phosphatase known to dephosphorylate IR [100]; (iv) inhibit both HMGCR by down-regulating the mevalonate (MVA) pathway and the antioxidant enzyme glutathione peroxidase 4 (GPX4), which are involved in the metabolic reprogramming-guided regulation of pro-tumorigenic transcription factors [101]; and (v) regulate the expression of genes influencing tumor suppression, apoptosis and angiogenesis via epigenetic mechanisms, including DNA methylation, histone acetylation and microRNA modulation [102]. In line with previous experimental evidence indicating that NUPR1 expression increases in the presence of high levels of reactive oxygen species (ROS) [69,103], the potential of statins to interfere with the anabolic processes triggered by insulin in cancer cells [104,105] remains to be investigated.

In the present study, the anti-proliferative action of statins via NUPR1 was demonstrated only by performing in vitro assays, which may not entirely reflect the biological heterogeneity and complexity of breast tumors encountered in vivo. However, it should be noted that alongside the widely recognized MCF7 cells, BCAHC-1 cells may serve as a valuable resource. This naturally immortalized primary cell line is distinguished as the only known BC cell model expressing IR in the absence of IGF1R. This peculiar characteristic allowed us to dissect the insulin effects mediated exclusively by IR, without interference from the IGF1R signaling, which is also insulin-responsive. Anyway, further validations in more physiologically complex settings are needed to extend our findings toward the rational repurposing of statins as adjunctive therapies in BC expressing high levels of IR. In this respect, it should be deemed that diverse issues can influence the intratumoral exposure and overall efficacy of statins, including hepatic metabolism, dosage selection, therapeutic time windows, patient stratification by BC molecular subtype and predictive biomarkers, and potential drug–drug interactions [88,91,106]. For instance, atorvastatin (80 mg/day) or simvastatin (40 mg/day), administered two weeks before surgery in patients with primary invasive BC, reduced tumor growth and proliferative markers, particularly in cancers expressing HMGCR [64]. Similarly, simvastatin exposure (20–40 mg daily for 2–4 weeks) in early-stage tumors increased apoptosis and cell cycle arrest [90]. On the contrary, in metastatic BC, simvastatin (40 mg/day) during chemotherapy did not show any significant improvement in the response or survival of ER-positive or triple-negative BC [107]. Collectively, these studies suggest notable differences in statin dosage and duration, as well as BC molecular subtype and stage. In this complex scenario, our findings showing that statins exert inhibitory effects on BC cells by targeting the insulin/IR/NUPR1 axis can provide novel insights supporting the need for additional randomized trials to evaluate the therapeutic efficacy of statins in ER-positive BC patients with high IR expression and/or elevated circulating insulin.

## 5. Conclusions

Here, we ascertained new molecular mechanisms through which different statins inhibit the proliferative effects mediated by insulin/IR signaling. Combined with the widespread clinical use of statins, our data further highlight the potential of these lipid-lowering molecules as attractive candidates for repurposing in oncology. This is particularly relevant for BC patients with metabolic disorders, such as obesity, hyperinsulinemia and enhanced insulin/IR signaling, that contribute to the progression of this malignancy.

## Data Availability

The data used to support the findings of this manuscript are available from the corresponding authors upon reasonable written request after the publication.

## Supplementary Materials

The following supporting information can be downloaded at: https://www.mdpi.com/article/10.3390/cells15030284/s1, Figure S1: Immunoblots of IR and IGF1R expression levels in BCAHC-1 and MCF-7 BC cells; Figure S2: Statins inhibit the mitogenic action of insulin by interfering with the IR/NUPR1 axis in MCF7 cells.

## Abbreviations

The following abbreviations are used in this manuscript:

ACTB	Actin beta
BC	Breast cancer
DAVID	Database for Annotation, Visualization and Integrated Discovery
DFI	Disease-free interval
ER	Estrogen receptor
ET	Endocrine therapy
GPX4	Glutathione peroxidase 4
HMGCR	3-hydroxy-3-methylglutaryl-coenzyme A reductase
HR	Hazard ratios
IGF1R	Insulin-like growth factor 1 receptor
IGFBP-7	Insulin-like growth factor binding protein 7
IR	Insulin receptor
IRS	Insulin receptor substrates
KEGG	Kyoto encyclopedia of genes and genomes
METABRIC	Molecular taxonomy of breast cancer international consortium
MVA	Mevalonate
NUPR1	Nuclear protein 1
PI3K	Phosphoinositide 3-kinase
PTPN1	Protein tyrosine phosphatase 1B
ROS	Reactive oxygen species
SERMs	Selective estrogen receptor modulators
TCGA	The Cancer Genome Atlas
