# Supplementary material for: Stress-Inducible Transcription Factor NUPR1 Is Involved in the Inhibitory Effects Exerted by Statins on Insulin Action in ER-Positive Breast Cancer Cells
Source: Cells. 2026 Feb 2;15(3):284. doi: 10.3390/cells15030284 (PMC12897195; doi:10.3390/cells15030284)
Supplement: Supplementary file 1 [file cells-15-00284-s001.zip › cells-4082534-supplementary.pdf]

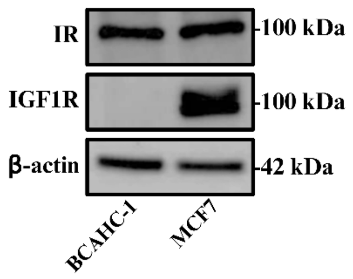

Supplementary Figure S1. Immunoblots of IR and IGF1R expression levels in BCAHC-1 and MCF-7 BC cells. Representative immunoblots of IR, IGF1R, and β-actin (which served as a loading control) from BCAHC-1 and MCF7 cells.

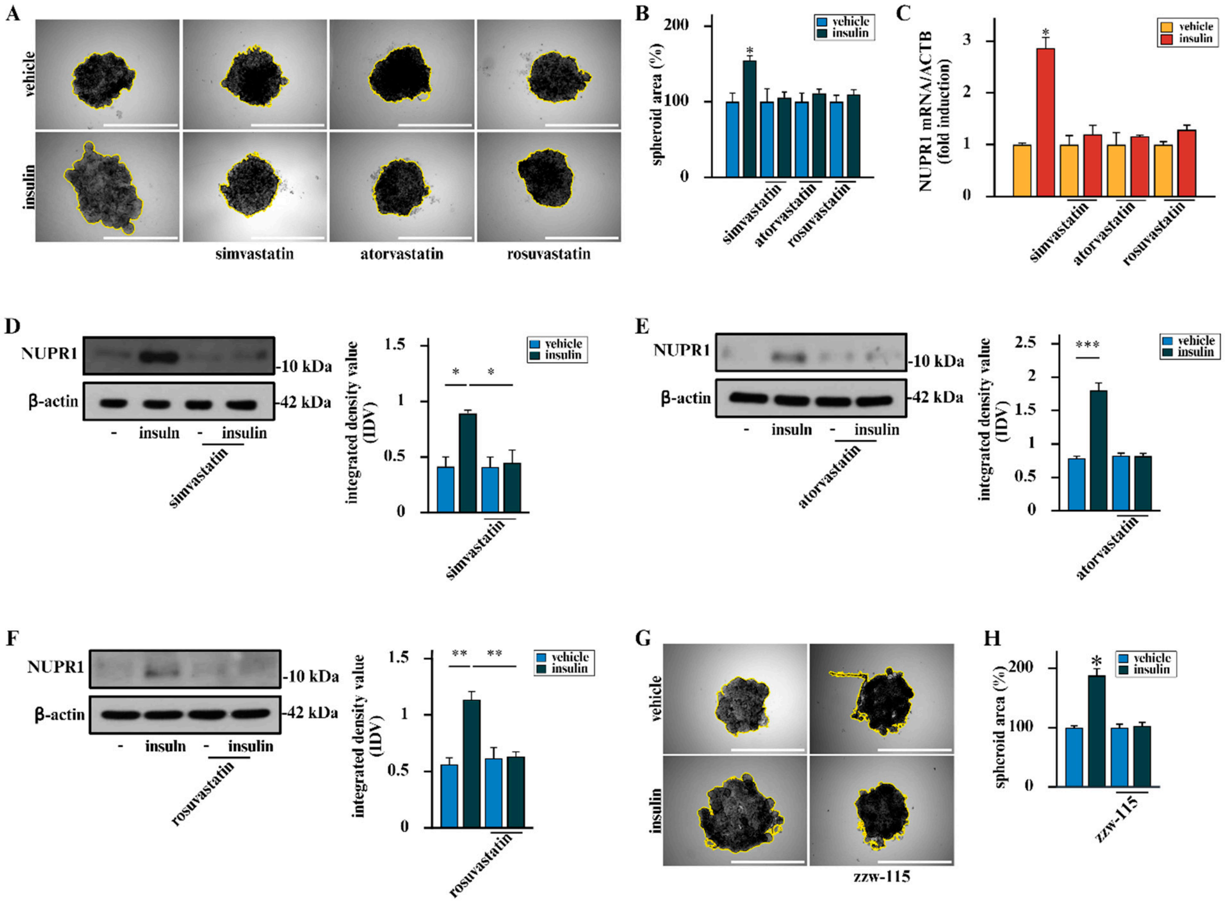

Supplementary Figure S2. Statins inhibit the mitogenic action of insulin by interfering with the IR/NUPR1 axis in MCF7 cells. **(A)** Representative pictures of spheroids (a single spheroid/well) from the MCF7 spheroid cultures grown on agar-coated plates and exposed for 10 days to vehicle or 10 nM insulin alone or in combination with 1 µM simvastatin, atorvastatin or rosuvastatin. Scale bar: 1000 µm. **(B)** Quantification of spheroid growth. Values of vehicle-treated MCF7 cells were set as 100% upon which spheroid growth was determined. **(C)** mRNA levels of NUPR1 evaluated by real-time PCR in MCF7 cells exposed for 6 h to vehicle or 10 nM insulin alone or in combination with 1 µM simvastatin, atorvastatin or rosuvastatin, which were added to the culture medium 18 h before vehicle or insulin exposure. Values are normalized to the actin beta (ACTB) expression and presented as fold changes of mRNA expression upon treatments relative to vehicle. Immunoblots of NUPR1 from MCF7 cells treated for 6 h with vehicle or 10 nM insulin alone or in combination with 1 µM simvastatin **(D)**, atorvastatin **(E)** or rosuvastatin **(F)**, which were added to the culture medium 18 h before the treatment with vehicle or insulin. Side panels show integrated density value (IDV) calculated as a ratio of NUPR1 to β-actin that served as loading controls, as indicated. **(G)** Representative pictures of spheroids (a single spheroid/well) from the MCF7 spheroid cultures grown on agar-coated plates and exposed for 10 days to vehicle or 10 nM insulin alone or in combination with 10 nM zzw-115. Scale bar: 1000 µm. **(H)** Quantification of spheroid growth. Values of vehicle-treated MCF7 cells were set as 100% upon which spheroid growth was determined. Values represent the mean ± SD of three independent experiments performed in triplicate. (*) indicates $p < 0.05$, (**) indicates $p < 0.005$, (***) indicates $p < 0.0005$, (****) indicates $p < 0.0001$.
